# Outpatient therapy with piperacillin/tazobactam using elastomeric pumps in patients with *Pseudomonas aeruginosa* infection

**DOI:** 10.1038/s41598-021-88179-7

**Published:** 2021-04-21

**Authors:** Jose Luis Lamas Ferreiro, Judith Álvarez Otero, Ana Sanjurjo Rivo, Lucía González González, Irene Rodríguez Conde, María Fernández Soneira, Javier Posada García, Javier de la Fuente Aguado

**Affiliations:** 1 Internal Medicine (Infectious Diseases Unit), Ribera Povisa Hospital, C/Salamanca 5, 36211 Vigo, Pontevedra Spain; 2 Internal Medicine, Ribera Povisa Hospital, Vigo, Spain; 3 Microbiology, Ribera Povisa Hospital, Vigo, Spain; 4 Hospital at Home, Ribera Povisa Hospital, Vigo, Spain

**Keywords:** Clinical microbiology, Antibiotics

## Abstract

The aim of this study was to evaluate the efficacy and safety of outpatient antimicrobial therapy with piperacillin-tazobactam in continuous infusion using elastomeric pumps and to evaluate the economic impact compared with conventional hospital treatment in patients with *Pseudomonas aeruginosa* (PA) infections. This is an observational study. Patients with PA infection treated with continuous piperacillin-tazobactam infusion using elastomeric pumps in our hospital between January 2015 and December 2017 were included. Primary outcomes were mortality during antibiotic treatment and mortality at 30 days. Secondary outcomes were reinfection or relapse at 30 days and clinical cure rate. The cost of each episode was compared with theoretical cost of the same treatment using conventional hospitalization. 35 patients were included. One patient (2.9%) died during the treatment. Overall 30-day mortality was 5.7%. No death was related to infection by PA. One patient (2.9%) had a reinfection at 30 days. Cure was achieved in 93% of patients at the end of treatment. There were no severe complications related to elastomeric pumps. Treatment cost with outpatient antimicrobial therapy was 67% lower than theoretical cost with conventional hospital treatment. Oupatient antimicrobial therapy with piperacillin-tazobactam in continuous infusion using elastomeric pumps in patients with PA infections is safe and effective with lower costs.

## Introduction

*Pseudomonas aeruginosa* is a non-fermentative gram-negative bacillus with intrinsic resistance to several antimicrobial agents. *Pseudomonas aeruginosa* infections are common in hospitalized patients and have a variable mortality at 30 days depending of the source of infection (from 7% in urinary tract infections to 48% in bacteremic pneumonia)^1,2^. Parenteral administration is the only option in a great number of cases and therapeutic options for outpatient care are limited. Moreover, the posology of most parenteral antipseudomonal antibiotics requires to be administered in several daily doses^3^. This makes it much harder to offer an intravenous outpatient therapy and requires in most cases a hospital therapy which expose the patients to risks related with hospitalization and increases healthcare costs^4^. Pharmacological characteristics of piperacillin/tazobactam in terms of stability and PK/PD properties allow a 24-h continuous infusion administration and makes it possible to replace it with just a daily visit^5^. There are several options for ambulatory administration of antibiotics, with a wide range of recommended devices^6^. Elastomeric pumps do not require to be connected to power sources, do not impair the patient's movements, and reduce the amount of nurse visits in the event of potential electronic alerts^7^. The aim of this study was to assess the effectiveness and safety of outpatient parenteral antimicrobial therapy (OPAT) in patients with *Pseudomonas aeruginosa* infections using piperacillin/tazobactam in continuous infusion with elastomeric pumps, as well as to assess potential savings in costs comparing with conventional hospital therapy.

## Materials and methods

This is an observational and retrospective study.

Inclusion criteria: patients infected with *Pseudomonas aeruginosa* and treated with piperacillin/tazobactam using OPAT with elastomeric pumps between January 2015 and December 2017.

Exclusion criteria: cases where the antibiogram showed *Pseudomonas aeruginosa* resistant to piperacillin/tazobactam or polymicrobial infection by other microorganisms resistant to that drug.

In our institution OPAT is administered by the hospital at Home department. Piperacilin/tazobactam is administered in patient home with a nurse daily visit using elastomeric pumps with 240 ml of capacity and flow rate of 10 ml/h. Peripheral lines or, if necessary, peripheral inserted central catheter were used for drug infusion.

The following variables were analysed: age, gender, comorbidity, source of infection, dosage, number of days of antibiotic treatment, complications related to the infusion device, and clinical evolution. The primary outcome related to the effectiveness of OPAT was mortality during the episode and at 30 days since OPAT started. The secondary outcomes related to the effectiveness and safety of the treatment were: clinical and microbiological cure rate, frequency of reinfection or relapse by the same microorganism at 30 days and complications or adverse events related to the infusion device. A cost saving analysis was also carried out by our analytical accounting department. The cost of outpatient admission for each patient was calculated taking into account the length of stay, medications and tests performed during follow-up by the hospital at home department. A new calculation was made using the theoretical costs of conventional hospital admission instead of the cost of OPAT. The cost savings in outpatient therapy were calculated by the difference between the two assumptions. Finally, we analysed the factors related to a worse evolution or a higher rate of complications related to the device.

Baseline characteristics are presented using medians for continuous variables and frequencies or percentages for categorical variables. Analysis of dichotomous variables was made using Chi-square test or bilateral Fisher's exact test. Quantitative variables were analysed using Student T test or Mann–Whitney U test when appropriated. Multivariate analysis was performed using binary logistic regression. Statistical significance was established with two-tailed p < 0.05.

All methods were carried out in accordance with relevant guidelines and regulations. The study was approved by Galicia Research Ethics Committee (code 2019/200) which waived the need to obtain written informed consent. All data were deidentified.

## Results

814 patients were treated with outpatient parenteral antibiotic therapy during the study period. 35 of them met inclusion criteria. 54% of them were male, and the median age was 74 years (range 30–94). Baseline characteristics of patients are shown in Table 1. The median of Charlson's comorbidity index was 2, and the median of Barthel's index was 20. The most frequently source of infection was respiratory (42.9%), followed by the skin and soft tissues (28.6%), urinary (25.7%), and bone and joints (2.9%). All patients were hemodynamically stable upon starting outpatient parenteral antibiotic therapy. The median of days on therapy with piperacillin/tazobactam administered by elastomeric pumps was 10 days (range 3–30). Only 1 patient (2.9%) died during therapy, and the cause of death was not infection related. Global mortality within 30 days amounted to 5.7%, and the causes of death were not related to the infection by *Pseudomonas aeruginosa*. Only 1 patient (2.9%) with COPD and respiratory infection had a relapse within 30 days with onset of respiratory symptons again and growth of *Pseudomonas aeruginosa* in sputum. Infectious symptoms were resolved in 93% of patients after treatment with piperacillin/tazobactam. With regards to the safety of the OPAT administration device, the only complications were the loss or extravasation of the intravenous line (8% of cases), which was replaced without any complications. None of the variables analysed was significantly associated with a higher risk of mortality. History of hemiplegia was associated with an increased risk of elastomeric pump related complications (OR 7.7; CI 95 1.6–36.8; P = 0–01). Treatment cost with OPAT was 67% lower than theoretical cost with conventional hospital treatment. The estimated global cost saving was 56.341 euros (equivalent to 68000 USD).

**Table 1:** Baseline characteristics.

Baseline characteristics		
Male sex	54%	
Age (years)	74 (range 30–94)	
Charlson comorbidity index	2 (range 0–6)	
COPD	28.6%	
Heart failure	5.7%	
Chronic renal insufficiency	14.3%	
Cognitive impairment	28.6%	
Barthel index	20 (range 0–100)	
Source of infection	Respiratory	42.9%
	Skin/soft tissues	28.6%
	Urinary	25.7%
	Bone and joints	2.9%

## Discussion

To our best knowledge, this is the first study assessing the use of piperacillin/tazobactam with elastomeric pumps in continuous infusion for the specific treatment of *Pseudomonas aeruginosa* infections. This therapy has been linked, in our experience, to a high rate of clinical cure, absence of mortality linked to the infection process, few adverse events linked to the infusion device and significant theoretical savings in costs, with an estimation of 67% when compared to conventional hospitalization. Previous studies on ambulatory intravenous antibiotic therapy with elastomeric pumps show results that support their use as well, with high effectiveness rates and few complications linked to the device. Some studies mention piperacillin/tazobactam, although they do not specify neither the type of infection that led to its administration nor the specific results of therapy with that antibiotic^8,9^. Other works mention the ambulatory use of piperacillin/tazobactam without specifying the administration method^10,11^. One study performed in Switzerland with 150 patients treated between 2013 and 2017 assessed outpatient antibiotic therapy in continuous infusion with elastomeric pumps. The clinical cure rate was 95% and the adverse event rate was 11%, none of them severe. In this work, only 12 patients were treated with piperacillin/tazobactam. Among them, one had a relapse which was cured with a second round of the same therapy^8^. In a study using data from the Spanish Registry of Outpatient Parental Antibiotherapy, between 2011 and 2016, 1463 nosocomial infections were treated with outpatient parenteral antibiotics, 22% of them with elastomeric pumps. *Pseudomonas aeruginosa* was responsible for 14% of the infections and only 10% of them were treated with piperacillin/tazobactam. The overall results, in terms of effectiveness, were favorable, with a mortality rate of 0.9%^9^. Another study based on the same registry and focused on exacerbation therapy of chronic obstructive pulmonary disease reported 562 patients treated at home with OPAT, 212 with *Pseudomonas aeruginosa* infections. 115 were treated with piperacillin/tazobactam, 27% with elastomeric pumps. The results were also favorable, with a mortality rate of 1.1% and only 2.7% of complications related to the venous catheter^12^. Regarding the economic costs, no study has specifically analyzed the use of elastomeric devices for this type of infections. Several previous reports have shown significant cost savings with the use of outpatient parenteral antimicrobial therapy compared to conventional hospitalization, with an estimated cost savings of 57–81%^4,13^.

Limitations of our study were the observational design and the small number of patients. Moreover, all patients of our study were treated by Hospital at Home department, which limits the generalizability of our results for other kinds of OPAT programs.

## Conclusions

Outpatient therapy with piperacillin/tazobactam in continuous infusion using elastomeric pumps is safe and effective with lower costs compared to conventional hospitalization for the treatment of *Pseudomonas aeruginosa* infections.

## References

[1] Gomila A, Carratalà J, Eliakim-Raz N, Shaw E, Wiegand I, Vallejo-Torres L (2018). Risk factors and prognosis of complicated urinary tract infections caused by *Pseudomonas aeruginosa* in hospitalized patients: A retrospective multicenter cohort study. Infect. Drug Resist..

[2] Morata L, Cobos-Trigueros N, Martínez JA, Soriano A, Almela M, Marco F (2012). Influence of multidrug resistance and appropriate empirical therapy on the 30-day mortality rate of *Pseudomonas aeruginosa* bacteremia. Antimicrob. Agents Chemother..

[3] Slavik RS, Jewesson PJ (2003). Selecting antibacterials for outpatient parenteral antimicrobial therapy: Pharmacokinetic–pharmacodynamic considerations. Clin. Pharmacokinet..

[4] González-Ramallo VJ, Mirón-Rubio M, Mujal A, Estrada O, Forné C, Aragón B (2017). Costs of outpatient parenteral antimicrobial therapy (OPAT) administered by Hospital at Home units in Spain. Int. J. Antimicrob. Agents..

[5] Voumard R, Van Neyghem N, Cochet C, Gardiol C, Decosterd L, Buclin T (2017). Antibiotic stability related to temperature variations in elastomeric pumps used for outpatient parenteral antimicrobial therapy (OPAT). J. Antimicrob. Chemother..

[6] Norris A, Shrestha N, Allison G, Keller S, Bhavan K, Zurlo J (2019). 2018 IDSA clinical practice guideline for the management of outpatient parenteral antimicrobial therapy. Clin. Infect. Dis..

[7] Oliver G (2016). Optimising patient safety when using elastomeric pumps to administer outpatient parenteral antibiotic therapy. Br. J. Nurs..

[8] Voumard R, Gardiol C, Pascal A, Arensdorff L, Cochet C, Boillat-Blanco N (2018). Efficacy and safety of continuous infusions with elastomeric pumps for outpatient parenteral antimicrobial therapy (OPAT): An observational study. J. Antimicrob. Chemother..

[9] González Ramallo VJ, Mirón Rubio M, Estrada Cuxart O, García Leoni ME (2017). Usefulness of Hospital at Home in nosocomial infections: advantages and limitations. Rev. Esp. Quimioter..

[10] Durojaiye OC, Bell H, Andrews D, Ntziora F, Cartwright K (2018). Clinical efficacy, cost analysis and patient acceptability of outpatient parenteral antibiotic therapy (OPAT): A decade of Sheffield (UK) OPAT service. Int. J. Antimicrob. Agents..

[11] Chambers ST, Basevi A, Gallagher K, Carswell-Moyna A, Isenman H, Pithie A (2019). Outpatient parenteral antimicrobial therapy (OPAT) in Christchurch: 18 years on. N. Z. Med. J..

[12] Ponce González MA, Mirón Rubio M, Mujal Martinez A, Estrada Cuxart O, Fiuza Pérez D, Salas Reinoso L (2017). Effectiveness and safety of outpatient parenteral antimicrobial therapy in acute exacerbation of chronic obstructive pulmonary disease. Int. J. Clin. Pract..

[13] Psaltikidis EM, Silva END, Bustorff-Silva JM, Moretti ML, Resende MR (2017). Economic evaluation of outpatient parenteral antimicrobial therapy: A systematic review. Expert Rev. Pharmacoecon. Outcomes Res..

